# Supplementary material for: Systematic review and meta‐analysis of bulk RNAseq studies in human Alzheimer's disease brain tissue
Source: Alzheimers Dement. 2025 Mar 5;21(3):e70025. doi: 10.1002/alz.70025 (PMC11881636; doi:10.1002/alz.70025)
Supplement: Supplementary file 1 — Supporting Information [file ALZ-21-e70025-s006.docx]

**PubMed search strategy:**

(nanostring OR "RNA isoform" OR transcriptome OR transcriptomic OR RNA-seq OR RNAseq OR "RNA microarray" OR "mRNA microarray" OR "RNA sequencing") AND (Alzheimer OR Alzheimer’s) AND (human OR man OR woman OR men OR women OR patient OR patients OR "homo sapiens")

**Scopus search strategy:**

( TITLE-ABS-KEY ( nanostring ) OR TITLE-ABS-KEY ( "RNA isoform" ) OR TITLE-ABS-KEY ( transcriptome ) OR TITLE-ABS-KEY ( transcriptomic ) OR TITLE-ABS-KEY ( rna-seq ) OR TITLE-ABS-KEY ( rnaseq ) OR TITLE-ABS-KEY ( "RNA microarray" ) OR TITLE-ABS-KEY ( "mRNA microarray" ) OR TITLE-ABS-KEY ( "RNA sequencing" ) ) AND ( TITLE-ABS-KEY ( alzheimer ) OR TITLE-ABS-KEY ( alzheimer's ) ) AND ( TITLE-ABS-KEY ( human ) OR TITLE-ABS-KEY ( man ) OR TITLE-ABS-KEY ( woman ) OR TITLE-ABS-KEY ( men ) OR TITLE-ABS-KEY ( women ) OR TITLE-ABS-KEY ( patient ) OR TITLE-ABS-KEY ( patients ) OR TITLE-ABS-KEY ( "homo sapiens" ) )

**Web of Science search strategy:**

(ALL=nanostring OR ALL="RNA isoform" OR ALL=transcriptome OR ALL=transcriptomic OR ALL=RNA-seq OR ALL=RNAseq OR ALL="RNA microarray" OR ALL="mRNA microarray" OR ALL="RNA sequencing") AND (ALL=Alzheimer OR ALL=Alzheimer’s) AND (ALL=human OR ALL=man OR ALL=woman OR ALL=men OR ALL=women OR ALL=patient OR ALL=patients OR ALL="homo sapiens")
